# A retrospective cross-sectional analysis of re-contact rates and clinical characteristics in diabetic patients referred by paramedics to a community diabetes service following a hypoglycaemic episode

**DOI:** 10.29045/14784726.2021.9.6.2.1

**Published:** 2021-09-01

**Authors:** Karl Bloomer

**Affiliations:** Northern Ireland Ambulance Service ORCID iD: https://orcid.org/0000-0002-7822-4528

**Keywords:** hypoglycaemia, paramedic, re-contact

## Abstract

**Aims:**

To report the re-contact rates and clinical characteristics of individuals referred to community diabetic teams following non-conveyance by HCPC paramedics.

**Methods:**

A retrospective cross-sectional study of routinely collected data by the Northern Ireland Ambulance Service HSC Trust of individuals referred to a community diabetic service following ambulance attendance and non-conveyance. Data were collected over a 3-month period with ambulance service re-contact and clinical data analysed.

**Results:**

418 emergency calls were identified as relating to hypoglycaemia with 169 referrals being made, a referral rate of 40.4%. Patients treated with insulin represented the majority of calls and tended to have a lower Glasgow Coma Scale score, but demonstrated many successful referrals. Increased age and multimorbidity were associated with repeat hypoglycaemic episodes and EMS attendance while other subgroups traditionally considered higher risk, such as patients with infections or under the influence of alcohol, showed potential for safe community management.

**Conclusion:**

The majority of paramedic referrals to community diabetic teams were successful, with less than 5% re-contacting the ambulance service within 3 days. This study, although demonstrating a lower referral rate than previous research, reinforces the safety of paramedic management and community referral for hypoglycaemia.

## Introduction

Hypoglycaemia, a state in which too little glucose exists in the blood, is a common complication resulting in EMS attendance for patients with diabetes. Villani et al. (2016) point out that reported figures of EMS attendance for hypoglycaemic episodes vary from 0.6 to 4.7% of total call volume. While the exact demand that hypoglycaemia places on ambulance services may still be uncertain, it is a condition that is likely to continue to grow in line with the increasing numbers of patients diagnosed with diabetes and subsequently starting treatment regimens.

Until recent years, UK ambulance services attending these patients would either transport patients to an emergency department or discharge at scene following successful treatment with little if any referral or community follow-up. Referral pathways were established as part of the ‘Transforming Your Care’ programme implemented by the Department of Health for Northern Ireland (2012) to ensure appropriate follow-up for patients suitable to be managed in the community and avoid unnecessary transportation to an emergency department. This was not a change unique to Northern Ireland and indeed was part of a UK-wide drive to develop ambulance service referral pathways (Snooks et al., 2004). These referral pathways were aimed at moving to a model that offered, where appropriate, care within the community and away from acute hospital settings; however, there remains limited evidence or validation to guide these referral pathways (Bell & Fitzpatrick, 2016; Department of Health, 2005).

## Objectives

This study sought to determine the level of community referral by paramedics in the Northern Ireland Ambulance Service (NIAS) and the subsequent number of patients re-contacting EMS following this. The study also sought to establish the clinical characteristics and demographics of those patients re-contacting EMS with the hope that this may influence practice and further research in hypoglycaemia management.

## Methods

The study was a retrospective cross-sectional design. It was conducted utilising data from the Emergency Ambulance Control (EAC) centre’s database, along with routinely collected clinical information on paper-based patient report forms (PRFs), and analysed using a quantitative methodology.

### Setting

This study was carried out using data collected across Northern Ireland from calls attended by the Northern Ireland Ambulance Service. In 2017 there were 92,480 adults (approximately 4.9% of the population) in Northern Ireland diagnosed with diabetes, with approximately 85% of these being type 2 (Northern Ireland Statistics and Research Agency, 2018).

### Study size

During the study dates of 1 April 2017 to 30 June 2017, NIAS received 53,144 emergency calls, with 418 of these identified as relating to a hypoglycaemic episode, around 0.8%. From these 418 hypoglycaemic calls, 169 referrals were made to community diabetic teams. The study size was limited to a 3-month period due to researcher resource and time constraints and therefore certain factors such as seasonal variations are not reflected in the data.

### Population and participants

The study data were collected from patients that presented with hypoglycaemia and were referred to a local community diabetic team. Re-contacts were identified if referred patients had a subsequent ambulance service attendance within 30 days. Some patients were excluded from a referral option from the outset, such as those below the age of 18 or those patients not known to have diabetes.

**Figure 1:**
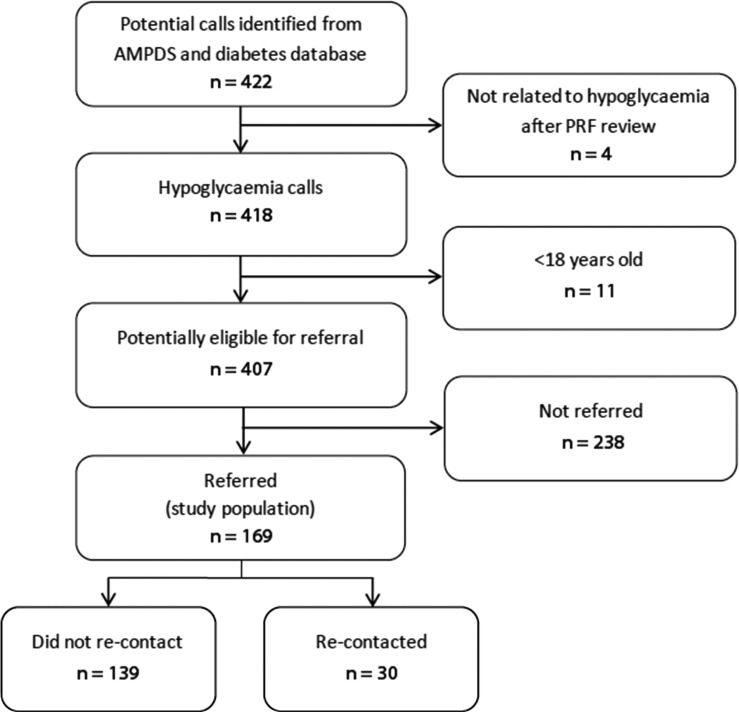
Figure 1. Flowchart for identification of study population.

Initial call volume was identified from the NIAS EAC database for calls coded as diabetic in nature by the Advanced Medical Priority Dispatch System (AMPDS) (Figure 1). Those patients that were referred and re-contacted the service were identified through a separate database for completed hypoglycaemic referrals where the original EAC unique incident number is used to link these to the emergency call. Identification of these participants was completed by the NIAS Quality Improvement team who process these data as part of their routine work.

### Data sources and measurement

PRFs that were identified using this method were reviewed in their anonymised form with 4 from a total of 422 calls removed due to being unrelated to hypoglycaemia. The true figures of hypoglycaemia-related calls are likely to be higher than those used for calculations of referral rates, as there will be a number of false negatives identified by AMPDS. Unfortunately, data cleaning for free-text and clinical observations from paper PRFs is not routinely carried out; therefore, ‘hypoglycaemia’ and blood glucose measurements were unavailable to be used as additional search criteria which would have perhaps identified additional hypoglycaemic patients that were not referred.

Clinical and non-identifiable demographic data were extracted from the anonymised PRFs by the researcher. Where the referral was not recorded as a ‘patient refusal to travel’ in the EAC database, the ‘refused transfer against clinical advice’ checkbox was not crossed, and where there was no mention of transfer refusal in the free text, this was recorded as a clinician-led referral decision for the purposes of data analysis.

### Bias

While some hypoglycaemia calls may not have been included in calculations for the referral rate, as detailed in the reasons above, selection bias will be low for this study as it is likely all eligible participants, i.e. those referred, were included. This is because referrals to community teams are captured by EAC at the time of the referral being made and are not subject to clinician error when recording on paperwork. However, there were a number of patients that were attended by the ambulance service in the study window that were neither transported to an emergency department nor referred, meaning more patients may have been eligible had clinicians referred these. Bias may also be introduced in the presented statistics as, due to missing data, available case univariate analysis was used to reach reported figures.

### Statistical methods

Descriptive statistics were used in the analysis and presentation of data, with particular attention paid to measures of central tendency and dispersion. Once data were gathered they were formatted to either a nominal or ratio level of measurement using Microsoft Excel. Analysed data were presented using frequency, percentages and central tendencies with reference to interquartile ranges. While inferential statistics would have been desirable, these were not possible due to resource and time limitations. Figures are presented at three re-contact time scales, <3 days, <7 days and <30 days, as this allows comparison with a wider range of previously published work which reported results at a number of different intervals (Bloomer, 2019).

## Results

### Participants

Due to local ambulance service referral procedures, only those patients of 18 years and over were eligible to be referred. During the study period, a total number of 418 calls were identified as relating to a hypoglycaemic episode; this was reduced to 407 (365 different patients) once patients <18 years were removed, with 169 referrals (the analysed data) made to the local diabetic community teams. This resulted in a relatively low referral rate of 41.5% when compared to other published figures (Bloomer, 2019). Sixteen referrals were for patients that had previously been referred during the study, meaning a total of 153 different patients were referred.

### Descriptive data

PRFs were available for all the patients that were referred; however, there were a number of inconsistencies across the recording of clinical and demographic data by clinicians, with areas – such as some clinical observations – occasionally incomplete. Where data were missing, figures were calculated using available-case analysis from recorded data. For diabetes disposition, the available data for statistics are recorded as n =. For clinical observations, at least 92% of possible data fields were available for all measurements except initial and final patient temperature, which ranged from 43% to 63% completion on PRFs depending on the re-contact timeframe.

### Overall re-contact rates

The data demonstrated a re-contact rate that was higher as time progressed. Patient-led refusals of transport which resulted in mandatory referral to community diabetic teams represented 26.04% (n = 44) of all referrals, with the rest (73.96%, n = 125) being clinician-led (Table 1). At all three periods of analysis (3, 7 and 30 days), clinician-led referral had a lower re-contact rate than patient refusals.

**Table 1.** Re-contact rates: referral and diabetes disposition.

	All referrals	Did not re-contact	Re-contacts <30 days	Re-contacts <7 days	Re-contacts <3 days
**Total figures**	169	139 (82.25%)	30 (17.75%)	13 (7.69%)	7 (4.14%)
**Clinician-led referrals 15 (3–21)**	125	106 (84.80%)	19 (15.20%)	8 (6.40%)	5 (4.00%)
**Transport refusals 12 (4–17)**	44	33 (75.00%)	11 (25.00%)	5 (11.36%)	2 (4.55%)
**Type 1 9 (4–19)**	47	37 (78.72%)	10 (21.28%)	5 (10.64%)	1 (2.13%)
**Type 2 18 (12–24)**	16	9 (56.25%)	7 (43.75%)	1 (6.25%)	1 (6.25%)
**IDDM 12 (4–19)**	151	124 (82.11%)	27 (17.88%)	12 (7.95%)	6 (3.97%)
**NIDDM 18 (1–21)**	9	6 (66.66%)	3 (33.33%)	1 (11.11%)	1 (11.11%)
**Sulphonylureas *Insufficient data***	6	4 (66.66%)	2 (33.33%)	0 (0.00%)	0 (0.00%)

Notes: Figures (in bold) re-contact median days (interquartile range); and n = (%).

### Diabetes type and treatment regimens

Available-case analysis was used when examining diabetic type and treatment regimens as not all PRFs recorded this information. Inconsistency existed in clinician recording of disposition, with many clinicians using the outdated terms of IDDM and NIDDM, instead of type. Therefore, data are presented with reference to both classifications so as to create a fuller picture, but also to allow for wider comparison with existing research, as older publications commonly reported exclusively under IDDM/NIDDM. There are cases where patients were analysed in more than one group – for example, where a patient’s PRF records type 2 but also a prescription of insulin – these were recorded under IDDM as well as type 2.

Of those patients with diabetes type recorded (n = 63), the majority were type 1 (74.60%, n = 47) and just over a quarter were type 2 (25.40%, n = 16). Type 2 diabetic patients had a higher 30-day re-contact rate compared to type 1 (43.75% vs. 21.28%), Insulin-dependent patients represented the largest identifiable group (n = 151) and re-contacted less frequently at all measured timescales. A limited number of patients (n = 6) were also identified as a subgroup prescribed sulphonylureas (Table 1).

### Patient age and comorbidities

The median age of those patients that were referred during the study period was 57 years. This was higher (62) in the re-contact group than for those that did not re-contact (54). The median age (66) was higher again when analysing those patients that re-contacted within 3 days. No one below the age of 30 (n = 15) re-contacted within 30 days following a referral (Table 2).

**Table 2.** Age demographics and comorbidities of re-contacts.

	All referrals n = 169	Did not re-contact n = 139	Re-contacts <30 days n = 30	Re-contacts <7 days n = 13	Re-contacts <3 days n = 7
**Age (years)**	57 (41.5–66.5)	54(41.0–66.0)	62 (46.0–68.5)	60 (46.5–68.5)	66 (47.0–71.0)
**Documented comorbidities (n)**	1 (0–2)	1 (0–2)	3 (1–4)	3 (1–4)	3 (2–4)

Notes: Figures presented as median and (interquartile range).

Those patients that re-contacted at any stage showed a higher number of comorbidities than those that did not require EMS attendance again. The most frequently reported comorbidity was hypertension (24.26%, n = 41) and for the purposes of calculations, diabetes was excluded when totalling comorbidities (Table 2).

### Clinical observations

The majority of clinical observations did not appear to show much fluctuation across the analysed re-contact points. The largest variance was that of initial Glasgow Coma Scale (GCS) score which showed a median of 14 for those that did not re-contact and decreased to 8 at <3 days (Table 3).

**Table 3.** Clinical observations in referred and re-contacting patients.

	All referrals n = 169	No re-contact n = 139	Re-contact <30 days n = 30	Re-contact <7 days n = 13	Re-contact <3 days n = 7
**Initial heart rate (beats/min)** **Final heart rate (beats/min)**	84.5 (73–98) 82 (71–92)	85 (73–99) 82 (71–92)	84 (72–93.8) 83 (70–92.5)	79 (69.5–102.5) 80 (66–103)	80 (70–105) 80 (64–109)
**Initial systolic BP (mmHg)** **Final systolic BP (mmHg)**	137 (123–153) 136 (123–152)	137 (120.5–153) 136 (120.8–152)	138 (128.5–160.5) 140 (124.8–157)	133 (119.5–151) 139 (122.5–154)	130 (111–183) 139 (121–183)
**Initial diastolic BP (mmHg)** **Final diastolic BP (mmHg)**	77 (71–87) 78 (70–87)	77 (71–87) 78 (70–87)	77 (67–85.8) 79.5 (69–87)	80 (63–84.5) 80 (71.5–92.5)	75 (59–91) 73 (65–95)
**Initial respiration rate (breaths/min)** **Final respiration rate (breaths/min)**	16 (16–18) 16 (16–18)	16 (16–18) 16 (16–18)	18 (16–20) 16 (15.5–18)	18 (16–21) 16 (16–18.5)	16 (16–20) 16 (16–18)
**Initial SpO2 (%)** **Final SpO2 (%)**	98 (96–100) 99 (97–100)	98 (96–100) 99 (97–100)	97.5 (96–99.8) 98 (97–100)	97 (96–99) 98 (96–100)	96 (93–99) 98 (93–100)
**Initial blood glucose (mmol/l)** **Final blood glucose (mmol/l)**	2.2 (1.6–3.1) 6.7 (5.4–8.5)	2.2 (1.7–3.3) 6.8 (5.4–8.8)	2.1 (1.6–2.8) 6.3 (5.2–7.7)	2.1 (1.7–3) 6.1 (5.2–8)	2.1 (1.6–2.3) 6.1 (5.2–7.8)
**Initial temperature (°C)** **Final temperature (°C)**	36.1 (35.2–36.6) 36.1 (35.2–36.5)	36.1 (35.1–36.6) 36.1 (35.2–36.5)	36 (35.5–36.5) 36 (35.3–36.5)	36.1 (35.9–36.3) 36.1 (35.9–36.3)	36.2 (35.9–36.7) 36.2 (35.9–36.7)
**Initial GCS score** **Final GCS score**	14 (10–15) 15 (15–15)	14 (11–15) 15 (15–15)	11 (7.8–14) 15 (15–15)	12 (8–14.5) 15 (15–15)	8 (7–12) 15 (15–15)
**Initial NEWS score** **Final NEWS score**	3 (2–5) 1 (0–2)	3 (1–4) 1 (0–2)	4 (3–5) 1 (0–2)	4 (2.5–5.0) 1 (0–2)	4 (4–5) 1 (0–2)

Notes: Figures presented as median and (interquartile range).

### Treatment

Glucagon was the most common treatment given to hypoglycaemic patients who were referred to diabetic community teams (39.64%, n = 67) and was also the most common treatment across all re-contact timescales when subgroup analysis was completed. The data revealed that those patients requiring greater levels of treatment re-contacted the ambulance service more often within the analysed timeframes (Table 4).

**Table 4.** Ambulance clinician treatment of referred and re-contacting patients.

	All referrals n = 169	No re-contact n = 139	Re-contact <30 days n = 30	Re-contact <7 days n = 13	Re-contact <3 days n = 7
**Treatment prior to EMS arrival**	42 (24.85)	38 (27.34)	4 (13.33)	1 (7.69)	1 (14.29)
**Food/carbs only**	37 (21.00)	34 (24.46)	3 (10.00)	1 (7.69)	0.00
**40% glucose gel**	61 (36.09)	45 (32.37)	16 (53.33)	4 (30.77)	2 (28.57)
**IM glucagon**	67(39.64)	48 (34.53)	19 (63.33)	7 (53.85)	5 (71.43)
**IV 10% glucose**	44 (26.04)	32 (23.02)	12 (40.00)	5 (38.46)	3 (42.86)
**IV 10% glucose dose in ml**	100 (100.00–150.00)	100 (100.00–150.00)	125 (100.00–187.50)	150 (125.00–200.00)	150 (150.00–200.00)

Notes: Figures presented as n = and (%); IV glucose volume presented as median and (interquartile range) ml {10 ml = 1 g}.

### Exclusion criteria and worsening care advice

There were five clinical backgrounds that JRCALC (2016) advised against non-conveyance for and had sufficient identifiable data for analysis. These were ‘complicating factors’; ‘under the influence of alcohol’; ‘signs of infection’; ‘recent hypoglycaemic episodes’; and ‘treated with sulphonylureas’. Three of these recommended conveyance groups showed a lower re-contact rate than the overall 30-day rate of 17.75%, whereas patients with recent hypoglycaemic episodes or those treated with sulphonylureas both showed a rate of 33.33% (Table 5).

**Table 5.** Re-contact details of patients fitting exclusion criteria and worsening care advice.

	Complicating factors n = 13	Alcohol n = 9	Infection n = 12	Recent hypos n = 12	OHA n = 6	WCA documented n = 103	No. WCA documented n = 66
**Re-contact rate (<30 days) n =**	2 (15.38%)	1 (11.11%)	2 (16.67%)	4 (33.33%)	2 (33.33%)	18 (17.48%)	12 (18.18%)
**Median (days)**	12.5	19.0	7.0	4.5	19.5	16.0	5.0
**Interquartile range**	n/a	n/a	n/a	3.3–5.8	n/a	6.5–22.0	2.3–16.5

Notes: OHA = oral hypoglycaemia agents; WCA = worsening care advice.

Of those records that either had specific detail of, or mentioned, that worsening care advice had been given (n = 103), 17.48% (n = 18) re-contacted within 30 days with a median time of 16 days. Those patient report forms that made no mention of worsening care advice at all (n = 66) showed a slightly higher re-contact rate of 18.18% (n = 12) but a notably shorter median re-contact time of 5 days (Table 5).

## Discussion

The study reported a referral rate of 40.43% by paramedics, with those patients that were identified as suitable for community management demonstrating consistently lower re-contacts than those that refused emergency department transport against advice. This reinforces findings in previous research that paramedics identify higher risk patients and advise emergency department conveyance. Additionally, the median time to re-contact was longer for clinician-led referrals (15 vs. 12 days), which meant those patients generally had a longer window for primary care follow-up and many subsequent episodes will have occurred even despite engagement with community diabetic teams.

Type 1 diabetes appeared to represent the overwhelming majority of encounters; however, clinicians in this study still seemed to prefer to record disposition according to insulin treatment, resulting in an incomplete data set. Type 2 patients more frequently re-contacted following ambulance discharge or referral, although this was not commonly in the acute phase, demonstrating still a window for community follow-up.

Insulin-treated patients tended to have a lower glucose result and a lower consciousness-level upon clinician arrival, consistent with previous findings (Parsaik et al., 2013; Sampson et al., 2017). Yet, despite this, these patients were more frequently treated at scene and managed by way of community referral.

Elderly patients in this study generally had an increased number of comorbidities (median age for no other comorbidities = 42.0 {IQR 30.0–62.3} vs. median age for ≥3 comorbidities = 58.5 {IQR 47.0–66.0}) and both these factors recorded increased re-contact rates. Comorbidities were common among those patients that acutely re-contacted, creating a picture of patients generally in poorer overall health experiencing severe hypoglycaemic episodes more often. Furthermore, those patients suffering from multiple comorbidities, particularly renal and hepatic impairments, will have a reduced ability to recover fully and quickly, and thus can be at risk of subsequent severe hypoglycaemic (SH) episodes or other complications, according to the wider literature (Cryer & Arbelaez, 2017; Seaquist et al., 2013).

Patients with lower blood glucose readings and a reduced GCS score had more acute re-contacts to the ambulance service, but caution should be used in interpreting this as there were many very severe hypoglycaemic episodes that did not re-contact. Previous studies also highlight the safety of pre-hospital treatment in insulin-dependent diabetes mellitus, despite this group consistently presenting in a more severe state of hypoglycaemia and more frequently (Holstein et al., 2003; Sampson et al., 2017; Walker et al., 2006). It seems inappropriate to use initial blood glucose readings, and to a similar degree initial GCS score, in isolation to guide pre-hospital transport decisions.

Khunti et al. (2013) demonstrated raised respiratory rate as a predictor for hospital conveyance, and while the authors state that the exact cause of this increase is uncertain, a higher level of co-morbidity or underlying infection may have been a contributor. Initial respiratory rate was slightly raised among those patients who re-contacted in this study, but further research is required to demonstrate if this could be predictive of non-conveyance re-contact.

Of those patients that re-contacted acutely, particularly in <3 days, glucagon was widely used for management despite the associated risks, such as depletion of glycogen stores which can contribute to recurrent episodes. Lower re-contact rates were noted among those patients treated prior to paramedic arrival, and treated less invasively. These patients may have been more aware of symptoms and warning signs and therefore able to seek help or corrective treatment earlier than those who had perhaps built up a degree of hypoglycaemia unawareness, or at least delayed awareness.

Forty-five patients were referred to diabetic community teams despite the patients fulfilling at least one recommended conveyance criteria (JRCALC, 2016). A recent episode of hypoglycaemia was the most common conveyance criterion for patients re-contacting (33.33%, n = 4) and also had a significantly lower median re-contact time than any other (4.5 days). While the findings seem to support excluding recent hypoglycaemic episodes from community management, other criteria appear to have the potential for allowing greater clinician flexibility. Underlying infections, complicating factors / certain co-morbidities and being under the influence of alcohol demonstrated lower re-contact rates and longer median re-contact times (7, 12.5 and 19 days respectively).

Prescription of sulphonylureas was only identified in six patients, of which two re-contacted. Further research including a larger number of patients would be required to address the continued uncertainty around the safety of these drugs and reoccurring hypoglycaemia in the pre-hospital setting and until then a risk-averse approach seems prudent.

Worsening care advice was not documented in nearly 40% of referred patients’ report forms and many more only documented that it was given but did not specify red flags or self-care over the coming hours and days. Even though it is likely that many more did receive the correct advice than was recorded, previous studies have highlighted the importance of written advice being issued following pre-hospital discharge and referral (Brooke Lerner et al., 2003; Cain et al., 2003; JRCALC, 2016). Given the potential vulnerability of this group and widespread service user knowledge gaps, specific written guidance or leaflets are recommended (Walker et al., 2006).

## Limitations

Some information, such as certain clinical observations, was occasionally missing from patient report forms and central tendencies had to be generated from what data were available. Furthermore, assumptions had to be made surrounding some clinician-led decisions due to the retrospective design of the study; these were applied consistently as a measure of control. Patient engagement with community referral services was outside this study’s scope and is a potential area for further exploration in future research.

Due to time constraints and data size, the period of analysis only included 3 months, therefore sessional variances in presentations and possible changes in re-contact rates were not examined.

## Generalisability

Future research examining larger population groups over a wider period would be needed to create a more complete picture of the effectiveness of community referral teams, particularly among patients with less common characteristics. Differences in EMS systems and scopes of practice will also limit the external validity of the findings.

## Conclusion

The study showed very limited numbers requiring ambulance attendance for a repeat episode in the acute stages following referral for hypoglycaemia. This is consistent with previous research that reported <3-day re-contact rates for non-conveyance of hypoglycaemia patients ranging from 2.1 to 9.0% (Mechem et al., 1998; Sinclair et al., 2018). The data also revealed a lower <3-day re-contact rate (4.14%) than average <24-hour re-contacts (~5%) for 10 of the UK ambulance services when analysing all presenting complaints (O’Cathain et al., 2018).

This study appears to further demonstrate that ambulance clinicians can treat and refer diabetic patients appropriately while identifying those requiring further assessment in emergency departments. The re-contact figures also suggested that there may be further subgroups that can be safely managed in the community as opposed to transporting to an emergency department, as currently recommended in certain guidelines. However, within these subgroups there are many patients with significant multi-morbidity and any EMS service managing these patients in the community would require clinicians with appropriate scopes of practice to match the complexity. Further research would be required to fully examine the safety of community management for certain, more complex subgroups discussed in this study.

## Acknowledgements

The author would like to thank Ciaran McKenna, Alison Vitty and the NIAS Quality Improvement team for their support.

## Author contributions

Karl Bloomer acts as the guarantor for this article.

## Conflict of interest

None declared.

## Ethics

This research project was granted ethical approval by the University of Cumbria’s Research Ethics Panel ref: 20 November 2017. The work was exempt from NHS/HSC REC review and informed patient consent as raw data were identified by the NIAS Quality Improvement team, who handle these data as part of their normal work, and any identifiable data were removed prior to receipt by the sole researcher (IRAS, 2018).

## Funding

None.
